# Probiotic *Lactobacillus rhamnosus* GG Enhanced Th1 Cellular Immunity but Did Not Affect Antibody Responses in a Human Gut Microbiota Transplanted Neonatal Gnotobiotic Pig Model

**DOI:** 10.1371/journal.pone.0094504

**Published:** 2014-04-10

**Authors:** Ke Wen, Christine Tin, Haifeng Wang, Xingdong Yang, Guohua Li, Ernawati Giri-Rachman, Jacob Kocher, Tammy Bui, Sherrie Clark-Deener, Lijuan Yuan

**Affiliations:** 1 Department of Biomedical Sciences and Pathobiology, Virginia-Maryland Regional College of Veterinary Medicine, Virginia Polytechnic Institute and State University, Blacksburg, Viginia, United States of America; 2 Department of Large Animal Clinical Sciences, Virginia-Maryland Regional College of Veterinary Medicine, Virginia Polytechnic Institute and State University, Blacksburg, Viginia, United States of America; University of Massachusetts Medical Center, United States of America

## Abstract

This study aims to establish a human gut microbiota (HGM) transplanted gnotobiotic (Gn) pig model of human rotavirus (HRV) infection and diarrhea, and to verify the dose-effects of probiotics on HRV vaccine-induced immune responses. Our previous studies using the Gn pig model found that probiotics dose-dependently regulated both T cell and B cell immune responses induced by rotavirus vaccines. We generated the HGM transplanted neonatal Gn pigs through daily feeding of neonatal human fecal suspension to germ-free pigs for 3 days starting at 12 hours after birth. We found that attenuated HRV (AttHRV) vaccination conferred similar overall protection against rotavirus diarrhea and virus shedding in Gn pigs and HGM transplanted Gn pigs. HGM promoted the development of the neonatal immune system, as evidenced by the significantly enhanced IFN-γ producing T cell responses and reduction of regulatory T cells and their cytokine production in the AttHRV-vaccinated pigs. The higher dose *Lactobacillus rhamnosus* GG (LGG) feeding (14 doses, up to 10^9^ colony-forming-unit [CFU]/dose) effectively increased the LGG counts in the HGM Gn pig intestinal contents and significantly enhanced HRV-specific IFN-γ producing T cell responses to the AttHRV vaccine. Lower dose LGG (9 doses, up to 10^6^ CFU/dose) was ineffective. Neither doses of LGG significantly improved the protection rate, HRV-specific IgA and IgG antibody titers in serum, or IgA antibody titers in intestinal contents compared to the AttHRV vaccine alone, suggesting that an even higher dose of LGG is needed to overcome the influence of the microbiota to achieve the immunostimulatory effect in the HGM pigs. This study demonstrated that HGM Gn pig is an applicable animal model for studying immune responses to rotavirus vaccines and can be used for studying interventions (i.e., probiotics and prebiotics) that may enhance the immunogenicity and protective efficacy of vaccines through improving the gut microbiota.

## Introduction

Gnotobiotic (Gn) pig models have been extensively used in studies of human rotavirus (HRV) because of the similar susceptibility to the Wa strain (G1P1A[8]) HRV infection and clinical manifestations in neonatal Gn pigs as in human infants [1]-[4]. Our previous studies demonstrated that probiotic *Lactobacillus acidophilus* NCFM (LA) at the appropriate dose was effective in reducing rotavirus diarrhea as well as enhancing immunogenicity of oral rotavirus vaccines in Gn pigs [5]. LA regulated rotavirus vaccine-induced immune responses in a dose-dependent manner in Gn pigs [5], [6]. Low dose LA (5 doses; up to 10^6^ colony-forming-unit (CFU)/dose) significantly enhanced effector T cell responses and down-regulated regulatory T (Treg) cell responses; high dose LA (14 doses; up to 10^9^ CFU/dose) significantly down-regulated effector T cell responses and up-regulated Treg cell responses [6]. Intermediate dose LA (9 doses, up to 10^6^ CFU/dose) significantly enhanced rotavirus-specific antibody secreting cell (ASC) and memory B cell responses induced by rotavirus vaccines in Gn pigs [5]. LA also dose-dependently regulated innate immune responses of cytokine producing dendritic cells and Toll-like receptor expressing antigen presenting cells [7]. The probiotic *Lactobacillus rhamnosus* (LGG) strain also enhanced the immunogenicity of rotavirus vaccines in Gn pigs at the appropriate dose in our previous study (Manuscript in preparation). LGG, but not LA has been evaluated in several human clinical trials as a vaccine adjuvant [8], including rotavirus vaccine [9] and influenza vaccine [10], therefore we chose LGG for the present study.

Interactions between probiotics and host immune responses can be influenced by gut microbiota, which is lacking in Gn pigs. A previous study of pigs showed that the gut microbiota composition in human gut microbiota (HGM) transplanted pigs resembles that from the human donor [11]. *Bifidobacteria* from human stool could not colonize the gut of HGM transplanted ex-germfree mice [12] but succeeded in colonization of HGM transplanted pigs [11]. Furthermore, the colonization and evolutional development of microbiota in the gut of HGM transplanted pigs was similar to that observed in humans [11]. Of most importance to human health, HGM transplanted pigs had a healthy gut immune system and similar immunity to pathogens as those in humans [13], which supports the applicability of HGM transplanted pigs in studying immune responses to human pathogens and vaccines. Therefore, to verify the functions of probiotic adjuvants demonstrated in the previous studies, the current study establishes a HGM transplanted neonatal Gn pig model of HRV infection and diarrhea to closely mimic the real context of rotavirus vaccination in infants and to test LGG's dose effects on the immune response profiles induced by the AttHRV vaccine.

## Materials and Methods

### Ethics statement

All animal experiments were performed in strict accordance with federal and university guidelines. Specifically, we adhered to the recommendations in the Guide for the Care and Use of Laboratory Animals of the National Institutes of Health and the American Veterinary Medical Association Guidelines on Euthanasia. The animal protocol was approved by the Institutional Animal Care and Use Committee at Virginia Tech (Protocol# 10-168-CVM and 13-187-CVM). Ethical Committee approval was received from Virginia Tech Institutional Review Board for the newborn human stool sample collection (IRB number 11-1049).

### Probiotic Bacteria and rotavirus

*Lactobacillus rhamnosus* GG strain (ATCC 53103) was used in the current study and propagated in lactobacilli MRS broth (Weber, Hamilton, NJ). LGG inoculums were prepared and titrated as we previously described [14].

The cell culture-adapted human rotavirus Wa strain (G1P1A[8]), derived from the 35^th^ passage in African green monkey kidney cells (MA104, ATCC# CRL-2378.1), was used as the AttHRV vaccine for inoculation of Gn pigs at a dose of 5×10^7^ fluorescent focus-forming units (FFU) [15]. AttHRV was also used as the detector antigen in the enzyme-linked immunosorbent assay (ELISA) [5] and as the stimulating antigen in the intracellular IFN-γ staining assay as described previously [16].

The virulent HRV (VirHRV) Wa strain was passaged through Gn pigs and the pooled intestinal contents from the 27^th^ passage were used to challenge Gn pigs at a dose of 10^5^ FFU. The median infectious dose (ID_50_) and median diarrhea dose (DD_50_) of the VirHRV in Gn pigs were determined as approximately 1 FFU [17].

### Human gut microbiota inoculum

Human gut microbiota was obtained from stool samples of a healthy male infant born in the United States who was delivered by C-section, solely breast-fed, and did not exhibit any signs of digestive disorders or receive any medication prior to stool sample collection. A freshly passed stool was diluted 20-fold and homogenized in sterile and pre-reduced 0.1 M potassium phosphate buffer (PBS, pH 7.2) containing 15% glycerol (v/v) to produce a 5% human fecal suspension, which was then aliquoted into 15 ml sterile tubes and injected with nitrogen for 1 min and stored at −80°C. After completing sample collection daily between 17 to 23 days of age, the fecal samples were then thawed, mixed and homogenized to make a large stock for the entire experiment. The stock was divided into 15 ml aliquots, injected with nitrogen, and stored at −80°C. In order to remove glycerol prior to feeding to piglets, the fecal samples were thawed and washed with at least ten-fold volumes of PBS, centrifuged at 2000 rpm/min for 10 min at 4°C, and then diluted to the original volume with PBS.

Death of neonatal pigs caused by opportunistic bacteria infection after transplanting HGM from an apparently healthy 11 years old human donor has been reported previously [18]. Therefore, before the study, the stool samples collected from the solely breast fed, healthy infant at 17–23 days of age were subjected to multi-step safety testing. First, the stool suspension was plated on blood agar plates to observe any sign of hemolytic activity which can be an indication of the presence of pathogenic bacteria. No hemolytic activity was observed. The stool sample was then screened by Viral Chips and the genomes were sequenced on an Illumina MiSeq™ at the Viral Diagnostics and Discovery Center, University of California, San Francisco. The sequencing results confirmed that no known viruses are present in this sample. Lastly, a bioassay was conducted. High doses of the stool inoculum (2.5-, 5-, and 10-times of the normal dosage of the stool inoculum) were tested in newborn Gn pigs and no adverse effect, except for mild diarrhea, was observed.

### Treatment groups and inoculation of Gn pigs

Gn pigs were derived and maintained as we previously described [6]. The pigs in the HGM groups were orally fed with 1 ml/dose of the 5% fecal suspension starting at 12 hrs after birth daily for 3 days to establish HGM transplanted Gn pigs. HGM transplanted Gn pigs were randomly assigned to five treatment groups: (1) Mock control with no treatment, (2) 14 doses of LGG (LGG14X) alone, (3) AttHRV alone, (4) AttHRV plus 9 doses of LGG (AttHRV+LGG9X), and (5) AttHRV plus 14 doses of LGG (AttHRV+LGG14X) (see Table 1 for the number of pigs for non-HGM group and HGM groups). Detailed probiotic dosing schedules for the intermediate dose (LGG9X) and high (LGG14X) dose were described in our previous publication [5]. Pigs from both non-HGM and HGM transplanted groups were vaccinated with the AttHRV vaccine following the same protocol as we previously described [6]. At post-inoculation day (PID) 28, subsets of HGM pigs from the three AttHRV groups and the non-HGM AttHRV group, all HGM pigs from the mock control and LGG14X groups were orally challenged with 10^5^ FFU VirHRV. After HGM inoculation from 0 to 12 of days of age and from postchallenge days (PCD) 0 to 7, pigs were examined daily for clinical signs (diarrhea) and fecal swabs were collected daily as we previously described [15]. Mononuclear cells (MNCs) were isolated from ileum, spleen, and peripheral blood of pigs euthanized on PID 28 or PCD 7 as we previously described [6], [15].

**Table 1:** Number of pigs in non-HGM group and HGM groups at beginning of study and at euthanasia on PID 28 and PCD 7.

Treatment groups	Total at PID 0	Excluded	PID 28	PCD 7
**Non-HGM group**				
AttHRV	18	0	6	12
**HGM groups**				
HGM only	4	1	0	3
HGM+LGG14x	4	0	0	4
HGM+AttHRV	12	4	4	4
HGM+AttHRV+LGG9x	13	3	6	4
HGM+AttHRV+LGG14x	10	1	4	5

### Detection of rotavirus and LGG shedding and assessment of rotavirus diarrhea

Rectal swabs were collected for 7 days after VirHRV challenge to assess rotavirus diarrhea and shedding. Pigs with daily fecal scores of ≥2 were considered diarrheic. Fecal consistency was scored as follows: 0, normal; 1, pasty; 2, semiliquid; and 3, liquid. Daily scores were added up for each challenged pig from PCD 0 to 7 to get the cumulative fecal score and then the average cumulative fecal score for all the pigs in each group was calculated. Virus shedding was detected by ELISA and cell culture immunofluorescence (CCIF) assay in processed rectal swab fluids as described previously [19], [20].

Feces and LIC were collected and processed as described previously [19], [20]. Bacterial DNA was extracted using the Wizard Genomic DNA Purification Kit (Promega, Madision, WI) according to the manufacturer's instruction. The extracted DNA was then used for enumerating LGG numbers by real-time PCR. Pure LGG with known numbers quantified as described in the previous study [14] was used as the standard to calculate relative numbers of LGG in the samples. Real-time quantitative PCR was carried out with RT^2^ SYBR® Green qPCR Mastermix (QIAGEN Inc., Valencia, CA). PCR cycling conditions were 5 min at 94°C, and then 40 cycles of 15 s at 94°C, 15 s at 60°C, and 30 s at 72°C. The reaction mixtures each contained 2 μl of the sample DNA and 18 μl of the master mix, which included the appropriate sense and antisense primers. The sense primer for LGG is 5′-CGCCCTTAACAGCAGTCTTCAAAT-3′ and the antisense primer is 5′-ACGCGCCCTCCGTATGCTTAAACC-3′ [21].

### Flow cytometry

The frequencies of specific subsets of effector and regulatory T cells were determined by using intracellular cytokine staining and flow cytometry as we previously described [6], [16]. Their absolute numbers were calculated based on their frequencies among MNCs and the total number of MNCs isolated from each tissue in ileum, IEL, spleen and blood of Gn pigs.

### Assessment of rotavirus-specific IgA responses in serum and small intestinal contents and IgG responses in serum

Sera were collected at PID0, PID13, PID22, PID28 and PCD7, and the small intestinal contents (SIC) were collected at PID28 and PCD7 to measure rotavirus-specific IgA and IgG responses using indirect isotype-specific antibody ELISA as previously described [22], [23].

### Statistical analysis

Kruskal-Wallis rank sum test was performed to compare all data, except for proportions of virus shedding and diarrhea among treatment groups, which were compared using Fisher's exact test. Statistical significance was assessed at p<0.05. All statistical analysis was performed using SAS program 9.2 (SAS Institute, INC, USA).

## Results

### Similar clinical signs in AttHRV-vaccinated pigs with or without HGM transplantation after VirHRV challenge

**Figure 1:**
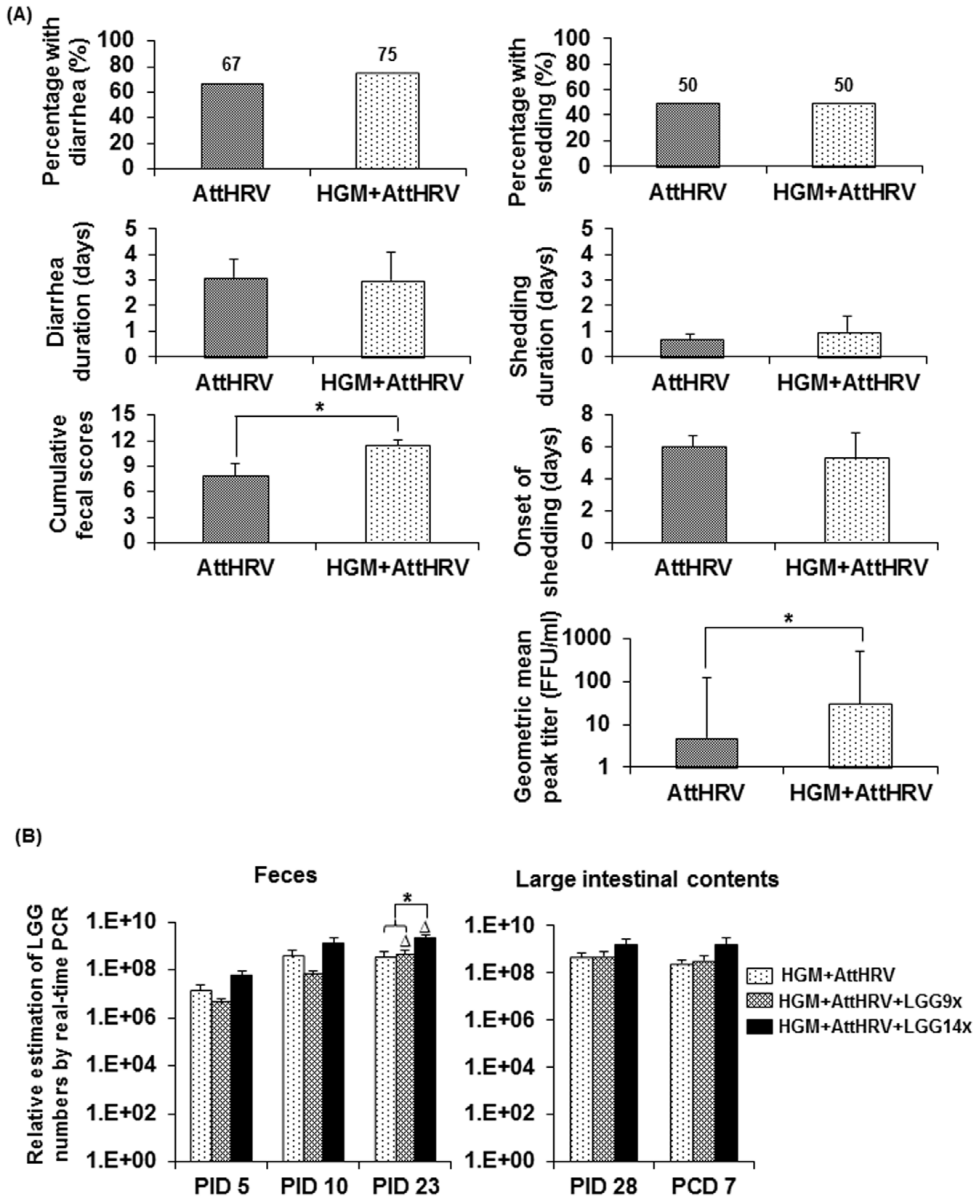
Clinical sign and virus shedding in AttHRV vaccinated pigs with or without HGM transplantation (A) and LGG shedding in fecal samples and large intestinal contents of HGM transplanted Gn pigs fed with or without LGG (B). After VirHRV challenge, pigs were monitored for 7 days for incidence of diarrhea, fecal score and virus shedding. Data are presented as mean ± standard error of the mean (n = 12 for AttHRV group; n = 4 for HGM+AttHRV group). The sign “*” in (A) indicates significant difference between groups (Kruskal–Wallis test, *p*<0.05). LGG amounts at different time points were determined by strain-specific real-time PCR and are presented as mean counts/ml ± standard error of the mean (n = 7–10 for fecal samples and n = 3–6 for large intestinal content samples). The sign “*” in (B) indicates significant differences between groups at the same time points and the symbol “Δ” indicates significant increases in LGG numbers compared to PID 5 for the same group (Kruskal–Wallis test, p<0.05).

Comparisons of clinical signs and fecal virus shedding of AttHRV-vaccinated pigs with or without HGM transplantation after VirHRV challenge are summarized in Fig. 1A. Compared to the Gn pigs, except for the significantly higher cumulative fecal scores and virus shedding titers in the HGM transplanted Gn pigs, both pig groups had similar incidence of diarrhea (67% and 75% for Gn and HGM pig groups, respectively) and fecal virus shedding (50% for both pig groups), similar mean duration of diarrhea and fecal virus shedding, as well as similar onset of fecal virus shedding. Both pig groups shed low titers of virus (<200 FFU/ml); however, the HGM pigs shed significantly higher virus titers (6-fold) compared to the Gn pigs (Fig. 1A).

### High dose LGG feeding significantly enhanced the fecal and intestinal LGG counts in HGM transplanted Gn pigs

Two LGG dosing regimens were included to investigate the effectiveness of different LGG feeding regimens in modulating the LGG counts in the feces and intestine of the HGM transplanted pigs (Fig. 1B). AttHRV+LGG14X pigs had significantly higher LGG titers in feces at PID 23 than AttHRV+LGG9X and AttHRV pigs; this high dose LGG feeding also resulted in higher (not significantly) LGG counts in feces at PID 5 and PID 10 and in large intestine contents (LIC) at PID 28 and PCD 7 compared to the other two groups. AttHRV+LGG9X and AttHRV only pigs had similar LGG counts from PID 5 to PCD 7. Since AttHRV only pigs were not fed LGG, the data indicate that the LGG detected in those pigs was a component of the donor HGM with successful colonization of the Gn pig intestine. Notably, the magnitude of increases of LGG counts in the AttHRV+LGG14X group (22-fold) was greater than the AttHRV+LGG9X group (14 fold) from PID 5 to PID 10, reflecting the difference in the LGG dosing regimens.

### HGM colonization significantly promoted the development of Th1 type immune responses and down-regulated Treg cell responses

**Figure 2:**
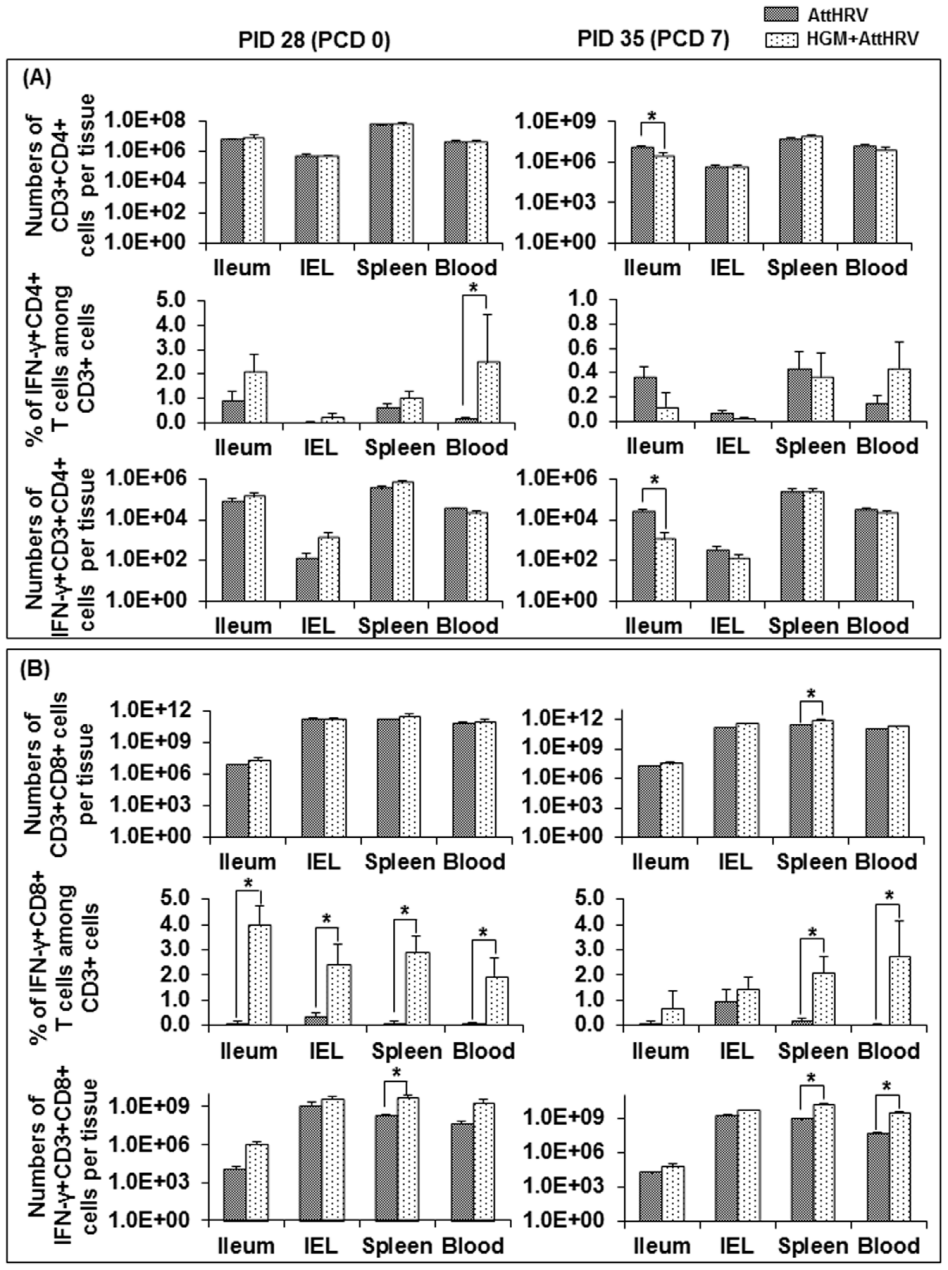
T cell responses in AttHRV vaccinated pigs with or without HGM transplantation. MNCs were stimulated with semi-purified AttHRV antigen *in vitro* for 17 hrs. Brefeldin A was added for the last 5 hrs to block secretion of cytokines produced by the T cells. HRV-specific IFN-γ producing CD4+ and CD8+ T cells was detected by intracellular staining and flow cytometry as we previously described [16]. The frequencies of IFN−γ+CD4+/CD8+ T cells were expressed as percentages among total CD3+ T cells (A and B, middle panel). All mean frequencies are reported after subtraction of the background frequencies. The absolute numbers of CD3+CD4+/CD8+ cells and IFN−γ+CD3+CD4+/CD8+ cells per tissue (A and B, top and bottom panels) were calculated based on the frequencies of CD3+CD4+/CD8+ cells and IFN−γ+CD3+CD4+/CD8+ cells, respectively, and the total number of MNCs isolated from each tissue. Data are presented as mean number or frequency ± standard error of the mean (n = 4–12). The sign “*” indicates the significant difference between groups (Kruskal–Wallis test, p<0.05).

**Figure 3:**
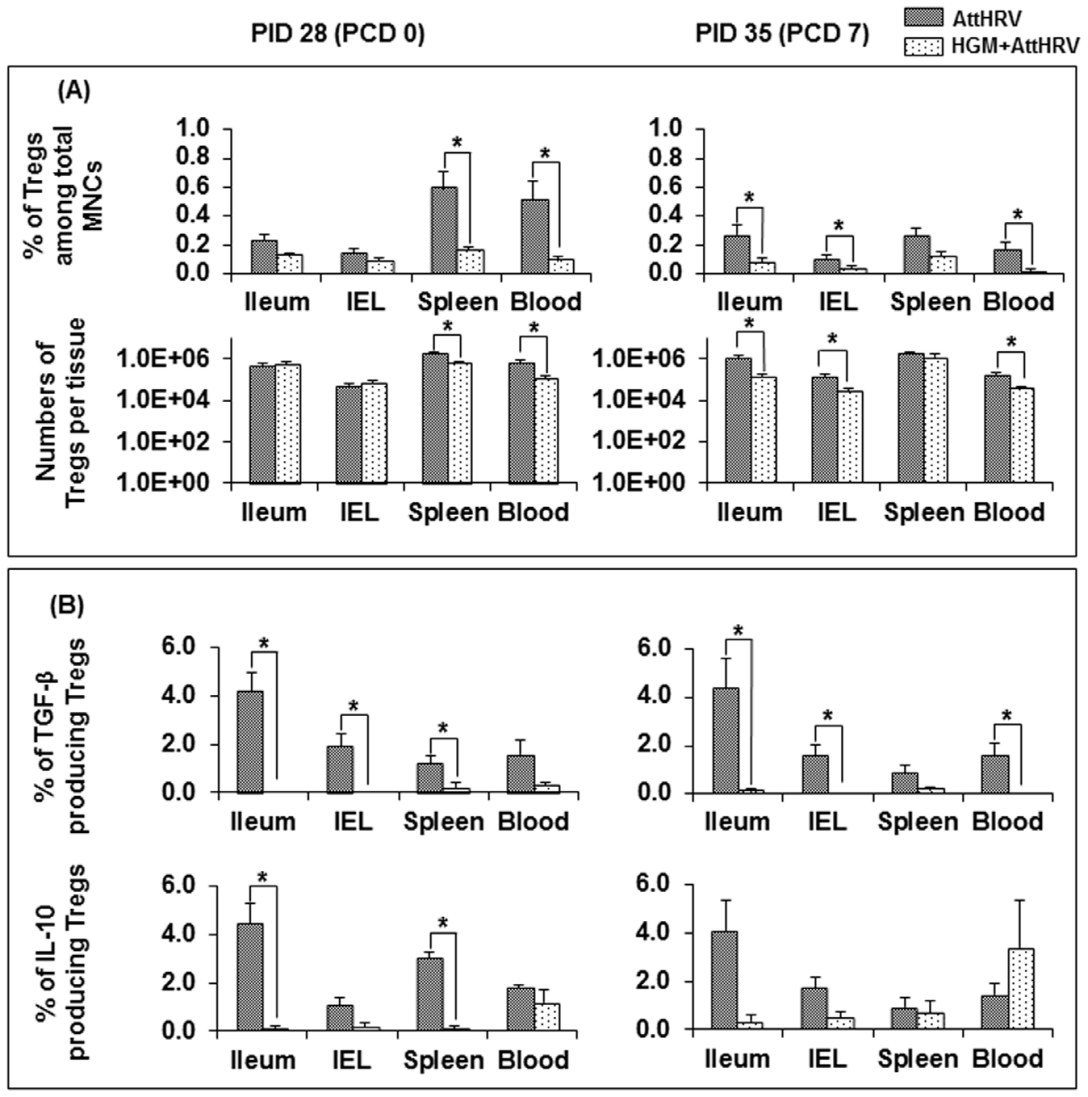
Treg responses in AttHRV vaccinated pigs with or without HGM transplantation. MNCs were stained freshly without *in vitro* stimulation. The frequencies of Tregs were expressed as the percentages among gated MNCs (A, top panel). The absolute numbers of Tregs per tissue were calculated based on the frequencies of Tregs and the total number of MNCs isolated from each tissue (A, bottom panel). The frequencies of IL−10+ or TGF−β+ Tregs were expressed as the percentages of IL−10+ or TGF−β+ cells among the Tregs (B). Data are presented as mean number or frequency ± standard error of the mean (n = 4–9). See Fig. 2 legend for statistical analysis.

To study interactions among HGM, neonatal immune system and rotavirus vaccine, and to evaluate the effect of HGM on the development of neonatal immune system in the context of rotavirus vaccination, we compared the total numbers and frequencies of effector (Fig. 2) and Treg cell (Fig. 3) subsets in the intestinal [ileum and intraepithelial lymphocytes (IEL)] and systemic (spleen and blood) lymphoid tissues of the AttHRV-vaccinated pigs with and without HGM. HGM did not significantly alter the total numbers of CD3+CD4+ and CD3+CD8+ T cells in any tissues tested at PID 28, suggesting that AttHRV provided sufficient antigen stimulation for the expansion of the T cell compartment of the neonatal immune system in Gn pigs without HGM. However, AttHRV-vaccinated pigs with HGM had significantly higher frequencies of IFN−γ+CD4+ T cells among CD3+ cells in blood and IFN−γ+CD8+ T cells among CD3+ cells in all tissues at PID 28 compared to AttHRV-vaccinated pigs without HGM. HGM+AttHRV pigs also had significantly higher numbers of IFN−γ+CD3+CD8+ T cells in spleen at PID 28 than AttHRV only pigs. Postchallenge, HGM significantly increased total numbers of CD3+CD8+ cells in spleen and significantly enhanced IFN−γ producing CD8+ T cell responses measured in both frequencies and numbers in spleen and blood of AttHRV-vaccinated pigs.

Consistent with the enhanced Th1 type immune responses, HGM down-regulated or significantly down-regulated the frequencies of CD4+CD25−FoxP3+ Treg cells in all tissues of AttHRV-vaccinated pigs. HGM+AttHRV pigs had significantly lower numbers of Treg cells in spleen and blood at PID 28 and in ileum, IEL and blood at PCD 7 compared to AttHRV only pigs. Furthermore, HGM decreased or significantly decreased the frequencies of IL-10 or TGF-β producing Treg cells in all tissues (with one exception in IL-10 producing Treg cells in blood at PCD7) of AttHRV-vaccinated pigs.

### High dose LGG significantly enhanced rotavirus-specific IFN-γ producing T cell responses but did not affect Treg cells in AttHRV-vaccinated pigs with HGM

**Figure 4:**
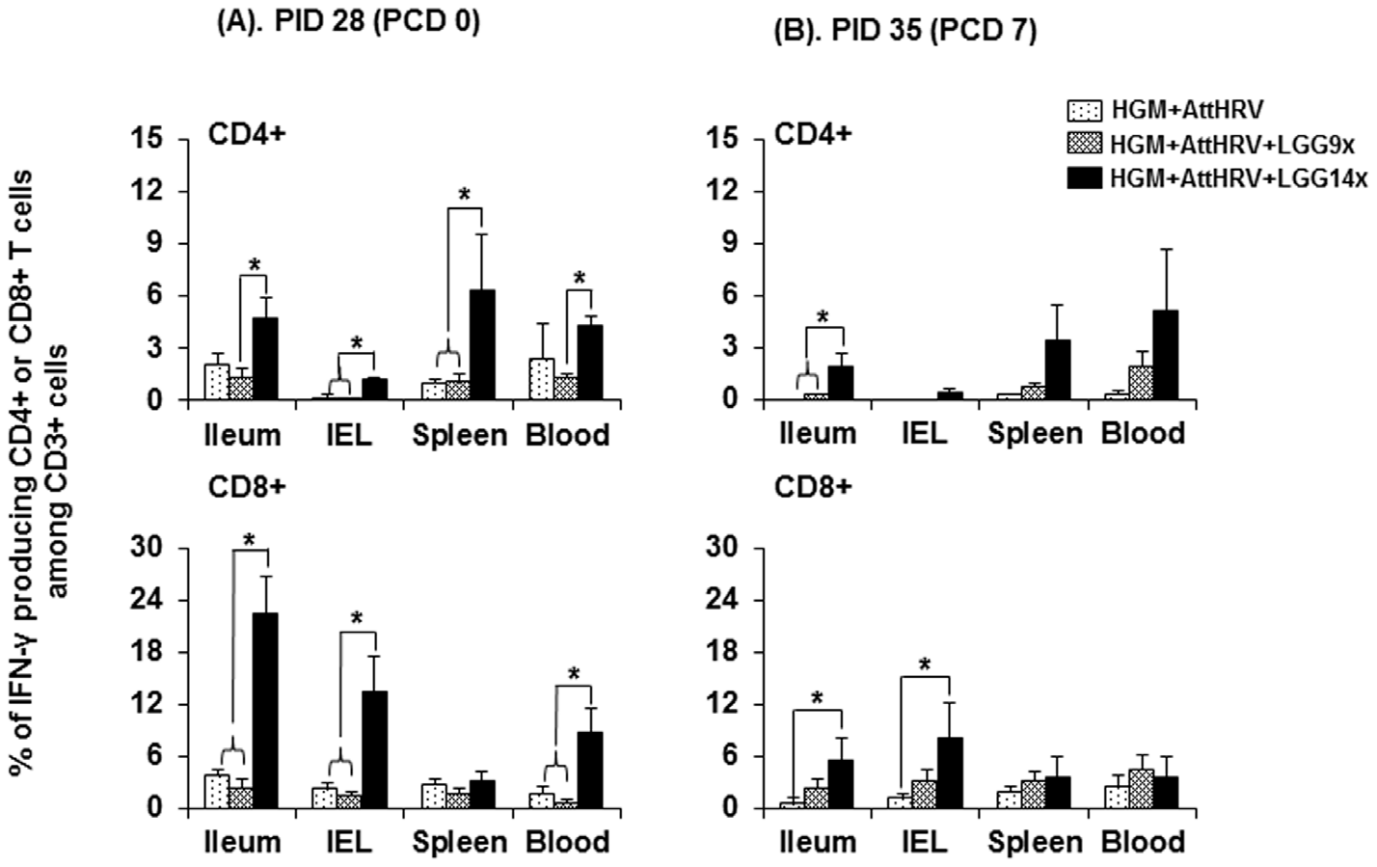
Rotavirus-specific IFN-γ producing T cell responses in HGM transplanted Gn pigs fed with different doses of LGG. Data are presented as mean frequency ± standard error of the mean (n = 4–6). See Fig. 2 legend for detection of rotavirus-specific IFN-γ producing T cell and statistical analysis.

To investigate the dose effect of LGG as an adjuvant in enhancing the immunogenicity of the rotavirus vaccine, we measured the virus-specific effector T cell responses in AttHRV-vaccinated HGM pigs fed with or without the 9 and 14 doses of LGG at challenge (PID 28) and postchallenge (PCD 7) (Fig. 4). The AttHRV+LGG14X pigs had significantly higher frequencies of rotavirus-specific IFN-γ producing CD4+ T cells in IEL and spleen and IFN-γ producing CD8+ T cells in ileum, IEL and blood compared to both the AttHRV and AttHRV+LGG9X pig groups at PID 28. The AttHRV+LGG14X pigs also had significantly increased frequencies of rotavirus-specific IFN-γ producing CD4+ T cells in ileum and blood compared to the AttHRV+LGG9X pigs at PID 28. Postchallenge, AttHRV+LGG14X pigs had significantly higher frequencies of IFN-γ producing CD4+ T cells in ileum than the AttHRV and AttHRV+LGG9X pigs. The AttHRV+LGG14X pigs also had significantly increased frequencies of rotavirus-specific IFN-γ producing CD8+ T cells in ileum and IEL compared to the AttHRV only pigs at PCD 7. The AttHRV+LGG9X pigs had slightly (not significantly) increased IFN-γ producing CD4+ and CD8+ T cell responses in all tissues at PCD 7.

No significant differences were observed in the frequencies of CD4+CD25−FoxP3+ Treg cells and the frequencies of their cytokine production in any tissues among the three AttHRV-vaccinated HGM pig groups with or without LGG feeding at PID 28 or PCD 7 (data not shown).

### Similar rotavirus-specific antibody responses associated with similar protection rate against rotavirus infection among all three groups of AttHRV-vaccinated HGM pigs with or without LGG feeding

**Figure 5:**
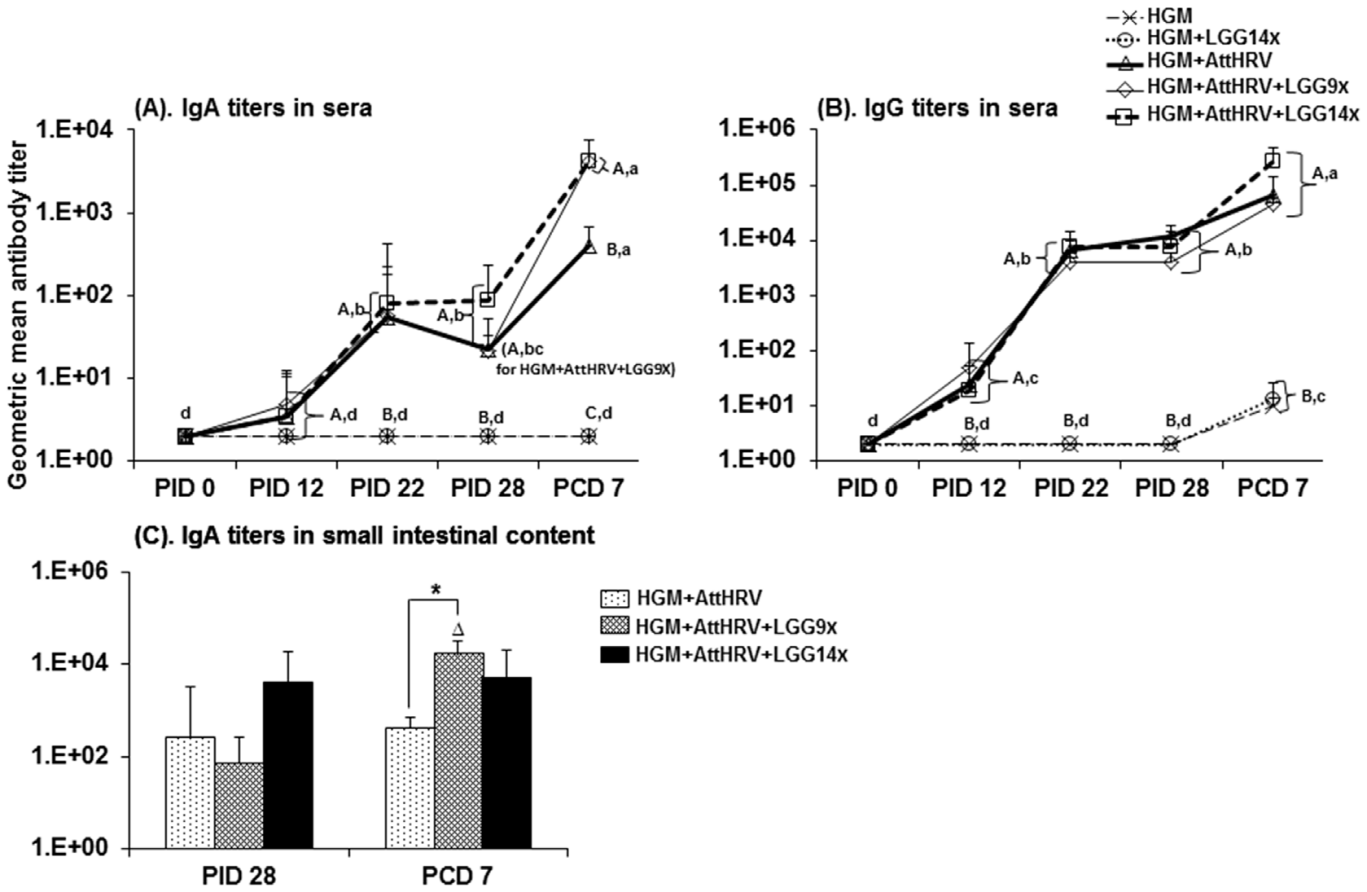
Rotavirus-specific serum IgA and IgG antibody responses (A and B) and rotavirus-specific IgA antibody responses in small intestine contents (C) of Gn pigs transplanted with HGM and fed different doses of LGG. Rotavirus-specific antibody titers were measured by an indirect isotype-specific antibody ELISA and presented as geometric mean titers for each treatment group + standard error of the mean (n = 3–10 for serum samples and n = 3–6 for small intestine content samples). Different capital letters (A, B, or C) indicate significant differences in antibody titers compared among different groups at the same time points; different lower case letters (a, b, c, d) indicate significant difference between different time points in the same group (Kruskal–Wallis test, p<0.05), whereas shared letters indicate no significant difference. The sign “*” indicates significant differences in IgA titers in small intestine contents between groups at the same time points and the symbol “Δ” indicates significant increases in IgA titers at PCD7 compared to PID28 for the same group (Kruskal–Wallis test, p<0.05).

In the HGM pigs, AttHRV vaccination with and without LGG induced significantly higher rotavirus-specific serum IgA responses at PID22, PID28 and PCD7 and serum IgG responses from PID12 to PCD7 compared to the unvaccinated pig groups (Fig. 5). Of interest, pigs from all three AttHRV groups with and without LGG feeding had similar rotavirus-specific serum IgA and IgG titers at each examined time point from PID0 to PID28 (Fig. 5). Consistent with this data, LGG feeding also did not significantly alter rotavirus-specific IgA responses in small intestinal contents at PID28, although AttHRV+LGG14X pigs had a trend for higher IgA titers than the AttHRV+LGG9X and AttHRV-only pigs at PID28 (Fig. 5). Postchallenge, however, the AttHRV+LGG9X and AttHRV+LGG14X pigs had significantly higher rotavirus-specific IgA titers in serum; AttHRV+LGG9X pigs also had significantly higher rotavirus-specific IgA titers in small intestinal contents compared to the AttHRV only pigs at PCD7 (Fig. 5). Serum IgG titers did not differ significantly among the three AttHRV groups at PCD7 (Fig. 5).

When analyzing the dynamics of antibody responses, pigs from all three AttHRV vaccinated groups had the highest rotavirus-specific serum IgA and IgG titers at PCD7, which were significantly higher than those examined at all previous time points (Fig. 5). All AttHRV pigs from the three groups also had significantly higher rotavirus-specific serum IgA and IgG responses at PID22 and PID28 compared to PID0 or PID12, and had significantly higher rotavirus-specific serum IgG titers at PID12 compared to those at PID0 (Fig. 5). Thus, LGG feedings did not significantly influence the magnitude and dynamics of rotavirus-specific antibody responses induced by the AttHRV vaccine in HGM pigs at PID28, but LGG enhanced the anamnestic antibody responses upon VirHRV challenge. The similar antibody responses were associated with similar corresponding protection against rotavirus fecal shedding observed in the three AttHRV pig groups upon VirHRV challenge (Table 2). There were no statistically significant differences in the percent of diarrhea and fecal virus shedding, mean days to onset and mean duration of diarrhea and virus shedding among the three AttHRV pig groups, likely due to the small number of pigs in each group. However, there was a trend for shorter mean duration of diarrhea and lower cumulative fecal scores in the LGG fed pigs, which is consistent with the moderate effect of LGG in reducing rotavirus diarrhea observed in Gn pigs [24] and in human infants [25].

**Table 2:** Clinical signs and rotavirus fecal shedding in AttHRV-vaccinated HGM pigs after VirHRV challenge.

Treatment groups	n	Clinical signs				Fecal virus shedding (by CCIF and/or ELISA)			
		% with diarrhea* ^,^ a	Mean days to onset^**^	Mean duration (days) ^**,^ b	Mean cumulative scores^**,^ c	% virus shedding*	Mean days to onset^**^	Mean duration (days)^**,^ b	Geometric mean peak titer (FFU/ml) ^**,^ e
AttHRV+LGG9x	4	100^A^	2.3 (1.3^d^)^A^	1.0 (0.0)^A^	9.6 (0.2)^B^	50^A^	5.5 (1.4)^A^	0.5 (0.3)^A^	7.5^A^
AttHRV+LGG14x	5	60^A^	4.0 (1.6)^A^	2.2 (1.1)^A^	10.3 (1.3)^AB^	60^A^	5.0 (1.3)^A^	1.2 (0.6)^A^	4.4^A^
AttHRV	4	75^A^	2.8 (1.8)^A^	3.0 (1.1)^A^	11.5 (0.6)^A^	50^A^	5.3 (1.6)^A^	1.0 (0.6)^A^	29.9^A^

a Pigs with daily fecal scores of ≥2 were considered diarrheic. Fecal consistency was scored as follows: 0, normal; 1, pasty; 2, semiliquid; and 3, liquid.

b For durations of diarrhea and virus shedding, if no diarrhea or virus shedding until the euthanasia day (PCD7), the duration (days) were recorded as 0 and the onset (days) were as 8 for statistical analysis.

c Mean cumulative score calculation included all the pigs in each group.

d Standard error of the mean.

e FFU, fluorescent focus forming units. Geometric mean peak titers were calculated among pigs that shed virus.

*Fisher's exact test or ** Kruskal-Wallis rank sum test was used for comparisons. Different letters indicate significant differences among treatment groups (p<0.05), while shared letters indicate no significant difference.

### Safety of the HGM in newborn Gn pigs

Although the multi-step safety testing was done to confirm the safety of the collected stool samples, 2–3 pigs in each litter became ill (poor appetite, lethargic, fever, vomiting, and/or diarrhea) during the experiments and had to be euthanized prior to the scheduled time point, despite treatment with 50% dextrose (8ml/dose) by mouth every 8 hrs. The mean mortality rate in the HGM pigs was 20.9% (Table 1). The necropsy reports from the Anatomic Pathology Laboratory, Virginia Tech Animal Laboratory Services, gave the final diagnoses as mild enteritis and mild hepatitis. The bacterial culture identified *Klebsiella oxytoca*, *Enterococcus faecalis*, and *Staphyloccus epidermdis* in the pigs. *Klebsiella oxytoca* was considered the likely primary pathogen. These early euthanized pigs were excluded from this study.

## Discussion

Gnotobiotic pigs offer distinct advantages for investigating enteric virus infections and vaccines and for dissecting immunomodulatory functions of probiotics. Gnotobiotic status prevents confounding factors from preexisting gut microflora and maternal antibodies that are present in conventionally reared animals or in humans. However, gnotobiotic animals also have some disadvantages. The major drawback is that the germ-free status causes underdeveloped intestinal lymphatic constituents and leads to the decreasing number of gut-associated lymphoid tissues [26]–[28]. Intestinal colonization of germfree animals with commensal microbes significantly promoted the development of mucosal and systemic immune systems [29]–[31]. Our current study demonstrated that HGM transplantation significantly promoted the activation of Th1 effector cells in both intestinal and systemic lymphoid tissues as evidenced by the increased numbers and significantly increased frequencies of IFN-γ producing CD8+ T cells in ileum, IEL, spleen and blood of the AttHRV vaccinated pigs at PID28. However, HGM did not significantly alter the total numbers of CD3+CD4+ and CD3+CD8+ T cells in any tissues of the AttHRV-vaccinated pigs at PID28, suggesting that exposure to the live AttHRV vaccine alone provided sufficient antigen stimulation for the expansion of the T cell compartment of the neonatal immune system in Gn pigs without HGM.

Our previous studies [5], [6] using Gn pigs demonstrated that probiotic *L. acidophilus* regulated T cell and B cell immune responses in a dose-dependent manner. We also found that the probiotic LGG dose-dependently regulated T cell and B cell immune responses in Gn pigs (manuscript in preparation). Due to competition between LGG and other bacterial species for colonizing the gut in HGM transplanted pigs, LGG doses higher than that used for Gn pigs were needed to effectively regulate immune responses in HGM pigs. We found that 14 LGG feedings (up to 10^9^ CFU/dose), but not 9 feedings (up to 10^6^ CFU/dose), enhanced virus-specific IFN-γ producing T cell responses in AttHRV vaccinated pigs transplanted with HGM; AttHRV+LGG14X pigs also had a trend for higher IgA titers than the AttHRV+LGG9X and AttHRV-only pigs at PID28, which suggests that an even higher dose of LGG may be needed to achieve the desired adjuvant effect in the HGM pigs [5]. In a recent study [32], Zhu et al. fed piglets with the total LGG (ATCC 7469) dose of 7×10^10^ CFU, which was called “low dose” but higher than the highest dose (total 2.2×10^9^ CFU) of LGG used in our studies. They found that 7×10^10^ CFU of LGG down regulated ileal IL-17A, enhanced ileal TGF-β1 and IL-10 mRNA but had no effect on IFN-γ, IL-12, and IL-4 mRNA expression in the small intestine [32], an immunoregulatory profile suggesting that the LGG dose was too high for an immunostimulatory profile desired by our purpose of using LGG as an adjuvant [5], [6]. Collectively, these studies suggest that all probiotic strains differentially regulate immune responses in each specific narrow dose range. Further studies are needed to identify the appropriate LGG dose and dosing regimen as a vaccine adjuvant to effectively enhance virus-specific antibody responses in the HGM pigs. The interplay between HGM, probiotics and host immune system is complex, yet it reflects the real-world situation of human infants vaccinated with rotavirus vaccines and treated with probiotics. The results from the HGM pigs provide more relevant reference for human clinical practice. However, using the HGM pig model, like human clinical trials, is more difficult than the Gn pig model to reach clear conclusions, and future experiments will be required to include a larger number of subjects in each treatment group to establish the correlations between T cell and B cell responses and protection.

Because Gn pigs derived by hysterectomy (unsuckled) are devoid of maternal antibodies due to the impervious nature of the sow placenta to immunoglobulin, it is expected that Gn pigs are highly prone to opportunistic pathogen infections from transplanted HGM. One early study reported that in two litters of HGM pigs, 17 of 24 died due to the opportunistic pathogen *Klebsiella pneumoniae* present in the HGM from an apparently healthy human donor of 11 years of age [18]. With these safety concerns in mind, the HGM inoculum we used in this study was screened carefully; however, 20.9% of the pigs still became ill within several days after the oral administration of the HGM from the infant donor. Bacterial cultures from the euthanized pigs identified *Klebsiella oxytoca* as the likely cause of the illness. To build safe inoculum pools of HGM from donor stools, a more comprehensive screening procedure is needed. In addition to the screening steps we performed in this study, high-depth and high-throughput sequencing analysis and species-specific PCR for detection of potential pathogenic bacterial species for pigs such as *Klebsiella* spp. should be performed.

AttHRV vaccination with or without HGM transplantation conferred similar levels of protection in Gn pigs against diarrhea, virus shedding, and overall clinical signs upon VirHRV challenge, even though HGM pigs had significantly higher Th1 type effector T cell responses and lower Treg cell responses compared to Gn pigs. Furthermore, high dose LGG feeding increased the fecal and intestinal LGG counts, suggesting that sufficient doses of LGG fed to pigs with preexisting HGM and prior colonization by LGG were able to further enhance the fecal and intestinal counts of LGG. High dose LGG feeding also significantly enhanced the intestinal and circulating virus-specific effector T cell responses, but did not significantly enhance intestinal or serum virus-specific antibody responses in HGM pigs at the time of challenge (PID28) and did not significantly enhance the protective efficacy of the AttHRV vaccine. The magnitude of virus-specific serum and intestinal IgA antibody responses induced by rotavirus vaccines is an important indicator of protective efficacy of the vaccines [5], [22]. The results from this study highlighted again the importance of antibody responses. It is worth noting that only the AttHRV+LGG9X pigs had significant increases in the intestinal IgA antibody response from PID 28 to PCD 7. The AttHRV+LGG14X pigs had significantly higher intestinal virus-specific IFN-γ producing CD4+ T cell responses than the AttHRV+LGG9X pigs at PID 28 and PCD 7, indicating that AttHRV+LGG14X pigs had a skewed Th1 type response in the intestine that suppressed the virus-specific intestinal IgA antibody responses. Nonetheless, there is a disagreement between the increased immunogenicity (as indicated by the increased level of intestinal IFN-γ producing T cells) of the AttHRV vaccine in the HGM over the Gn pigs and the similar levels of protection upon VirHRV challenge. A plausible explanation is that certain strains of bacteria in the HGM facilitated the replication of VirHRV in the gut leading to increased challenge virus load. We noticed that the mean peak virus shed in feces postchallenge in the HGM pigs were 6-fold higher than the Gn pigs. Our study of a porcine epithelial cell line (IPEC-J2 cells) showed that treatment of the cells with *L. acidophilus* prior to rotavirus infection significantly increased the amount of rotavirus antigens and the virus titers [20]. In two other previous studies, Gn pigs inoculated with the VirHRV and fed with *L. acidophilus* plus *L. reuteri* or inoculated with the AttHRV and fed with *L. acidophilus*, shed higher titers of the viruses in feces than the pigs not receiving lactobacilli feeding [33], [34]. Several other studies have also shown that gut microbiota enhanced the replication or virus entry of enteric viruses and their pathogenesis [35]–[37] and that elimination of microbiota delayed rotavirus infection and significantly reduced rotavirus infectivity in mice [37].

In previously reported studies using HGM pigs, HGM from a healthy 3-month old baby [38] or older human donors at 10, 14, 27–28, or 50–70 years of age [11], [13], [18], [38], [39] were used to transplant gut microbiota to newborn pigs. Considering the dramatic shifts in composition and diversity of the fecal microbiota that occur with age from infancy to adulthood [40], our HGM transplanted pig model more closely mimics the colonization and evolution of HGM in infants from a very early age and is a more applicable model for studying HRV, which causes gastroenteritis in infants.

In conclusion, both Gn pig and HGM Gn pig models are applicable models for the evaluation of rotavirus vaccines and therapeutics, but each model has its advantages and drawbacks. The Gn pigs have the advantage of being devoid of confounding factors from the gut microbiota, whereas HGM Gn pigs have the advantage of more closely mimicking real-world situations. This current study demonstrated that HGM Gn pig model is an appropriate model for studying HRV infection and vaccines and for evaluation of the immunomodulatory effects of probiotics. To the best of our knowledge, this is the first study to establish the neonatal HGM transplanted Gn pig model of HRV infection and immunity. The findings also have implications in using Gn animal models with HGM for studies of other pathogens and diseases.
